# Foreign

**Published:** 1841-07-03

**Authors:** 


### FOREIGN.

*MM. Vidal, Chelius, and Liston, on Erysipelas.*—Of all the diseases which come under the province of the surgeon, there is perhaps no one which deserves his more serious attention than *erysipelas:* whether viewed as a local or constitutional affection; that is, in relation to its topical or general effects; whether appearing in an idiopathic form, or as a sequence of injury, there are few diseases which require more accuracy of discrimination in the employment of general remedial means, or promptitude and decision in the adoption of the necessary local treatment for arresting the destructive ravages which almost inevitably follow the unchecked progress of the affection in its severer forms. We shall therefore devote the space which remains to us in our present article, to discussing and contrasting the relative merits of the various plans of treatment, local as well as general, which are advocated and adopted by our authors, and also by others, in this disease: but first of all we must define what we mean by erysipelas. The difficulty of precisely defining this disease appears to arise principally from the question whether all idiopathic forms of simple diffused inflammation of the skin should be included under the generic title of erysipelas, or whether this term should not be limited to spreading inflammation of the skin and subcutaneous cellular membrane. The definition which Mr. Cooper gives is that which is ordinarily accepted, namely, that "erysipelas is a cutaneous inflammation attended with redness, which disappears and leaves a white spot for a short time after being touched with the end of the finger; and the affection, which is irregularly circumscribed by a defined line, is characterized by a remarkable propensity to spread."*

It is thus that the modification termed erythema, or as we may call it, simple idiopathic cutitis, is included in the above definition, (which it is clear will apply equally to both affections,) an arrangement that is objectionable on account of the difference of circumstances under which each form arises, the accompanying constitutional derangement in true erysipelas, and the several tendencies of the two complaints. With this preliminary remark, we shall be content to follow our authors in the divisions which they adopt. Vidal alone gives a separate short section to the consideration of erythema. The various species of the genus erysipelas are differently given by different authors; indeed, the classifications are nearly as numerous as writers: thus Pearson has three forms, phlegmonous, œdematous, and gangrenous; Lawrence describes erythema, simple, phlegmonous, and œdematous erysipelas; Rust and Chelius again have but two divisions, namely, true and false; whilst Rayer has no less than seven forms. It is of very little import which of these classifications is followed, but if we were disposed to give a preference, it would be to one based on different principles to any of those above enumerated, and having reference to the exciting cause rather than to the actual condition of the affected structures; as, for instance, the idiopathic or primitive, the symptomatic or secondary, and the accidental: this distinction has been recognized by some authors. The *causes* of erysipelas may be referred either to the predisposition and existing condition of the constitution, or to the infliction of some local injury; the latter may however be said to involve the former, as we may recognize in the occurrence of this form of spreading inflammation after a wound, an evidence that there existed a predisposition to the disease, resulting generally from irregular living, or a coincident morbid condition of system which favored its development. The immediate exciting cause of traumatic erysipelas may, however, be of a simpler nature, such as the employment of improper dressings to wounds, or mechanical irritation, but the abuse of good living is the most rife of remote or predisposing causes: in no class of persons is this disease so much to be dreaded as amongst our brewers' laborers in town: it almost invariably takes on its severest form in these gross subjects, destroying limbs and frequently life. The influence of certain moral affections is also enumerated amongst the exciting causes of the disease, and M. Vidal cites an amusing example of a lady whose choleric days were always marked by erysipe-

* Dr. Willan includes erysipelas under the head of "Bullæ," on account of the appearance of vesications in many instances: it is more correctly classed with the Exanthemata, by Rayer.

las of the nose. Amongst the more rare forms of this affection, we may mention the erratic and metastatic, the latter not unfrequently proving fatal by attacking the brain or its membranes. Derangement of the uterine functions, either in the form of dysmenorrhœa or amenorrhœa, is sometimes connected with erysipelas. but probably the most uncommon type is that of *general* inflammation of the whole body.— Rayer quotes a case from Renauldin, where "the whole skin of the trunk and of the limbs, slightly swollen, presented a very intense erysipelatous blush; the face was the part least affected; the patient, who fell as if consumed by fire, was soon restored by the use of aperients and tepid baths frequently repeated."* Mr. Cooper also mentions a case of which he was informed, "where the attack was both periodical and universal,effecting a lady several times, at intervals of two years."

The most usual termination of erysipelas is by resolution and subsequent desquamation, sometimes in suppuration, and more rarely in gangrene; when either of the two latter supervenes it is an evidence of functional derangement of important organs, or of a debilitated and vitiated constitution. Circumscribed collections of matter are comparatively rare, but there is occasionally a partial and trivial suppuration in the subcutaneous cells after simple erysipelas, which must not be confounded with the more extensive and diffused formation of pus succeeding the severer form of phlegmonous inflammation of the cellular membrane: this latter has been named by Mr. Liston, "diffuse cellular infiltration," the sloughing which often succeeds, being ascribed by him to "the infiltration of acrid sanious matter into the subcutaneous cellular tissue round a wound or sore;" the destruction of surface in these cases is generally very rapid, and rarely to be arrested even by prompt treatment. On the disputed question of the contagious nature of erysipelas we have little to say; indeed we believe the contagionist party is but small in the present day. In his very interesting and practical paper on erysipelas, in the Med. Chir. Trans. vol. xiv., Mr. Lawrence expressed himself in favor of its contagious character; an opinion formerly advocated by Drs. Wells and Stevenson, and more recently and strenuously by Dr. Williams, of St. Thomas' Hospital; whilst Rayer and others very positively deny it. We are not disposed to attach much importance to the experiment mentioned by Dr. Williams in his work on Cutaneous diseases, namely, "that if a person be inoculated with the fluid contained in the phlyctenæ of a genuine erysipelas, a red painful diffusive swelling is produced." Certain it is that the disease will attack many patients at the same time, or in succession, spreading from ward to ward in an hospital; and whatever theory he may adopt, the practical surgeon knows too well the danger of operating under these circumstances, where his patient would be exposed to the influence of this justly dreaded disease; but we believe that in these instances, it is rather to be regarded as epidemic than contagious; and its restriction to some particular locality may be explained by casualties which at the time might elude detection.

The *treatment* of erysipelas has been very various: we can only notice some of the more prominent plans. In the milder forms of erysipelas, there is room for little dispute as to the proper course of treatment to be pursued; indeed, in many cases, nature is best able to manage for herself without interference, or with simple attention to diet, and the state of the bowels. Where there is much disorder of the digestive organs, and of the liver in particular, both Chelius and Liston recommend the early exhibition of emetics. "They are productive of but little good," the latter observes, "in the more advanced stage, and their place is advantageously supplied by nauseating doses of antimony, combined or not with purgatives." This surgeon further adds that he has found great benefit from the combination of gray powder and Dover's powder, when the tongue is dry and covered with a brown crust; and that saline purges are sometimes desirable. Rayer advocates the employment of mild diluents in the slighter cases, and adds that lotions with tepid or cold water, or with any mucilaginous decoction, will in general suffice to arrest the complaint. Where the inflammation is quite confined to the surface, Mr. Liston thinks the employment of lunar caustic admissible, and even desirable, as diminishing the local uneasiness, and sometimes arresting the extension of the disease; free puncturing with a fine lancet, not reaching beyond the vascular layer, is also practised by him in these cases, as a means of relieving the over-distended vessels, and giving vent to the serous effusion, if there be any; this plan is peculiarly appropriate where larger wounds would leave unseemly scars, as in erysipelas of the face and head: the relief afforded to the patient is, moreover, very considerable where these parts are affected.

Before passing on to the very important consideration of the general and local treatment of the severe forms of erysipelas, including phlegmon, it may be well to clear the way a little by pointing attention to the different theories and explanations of phenomena which have induced practitioners to arrive at such very opposite conclusions in their treatment of this disease. The adoption of any particular plan of treatment, and its indiscriminate application in all cases of even the most uniform complaint, cannot be too much deprecated; the systematic aiming at specifics, as an exclusive object in medical practice, cannot but mislead enthusiasts into the bye-paths of quackery, and retard the advancement of medical science;

* Willis's Rayer, p. 128.

there are other, and we conceive higher objects of generalization in the treatment of disease than the discovery of nostrums; and these will be found in the establishment of leading principles to which *symptoms* may be referred, and on which they may be treated: not that we mean to deny that we must all contend, to a certain extent, with disease on empirical principles; but we mean to assert that the specific treatment of symptoms classified in the way to which we have alluded, is not only more philosophical and successful, but is much more likely to improve the art, as well as elevate the science of medicine. Our present subject offers an apt illustration of the inutility as well as danger of attempting to lay down certain fixed principles for the cure of a formidable and frequently fatal disease; what is the practical result of these theoretical systems, and what are the rules determined by those who advocate them? One party says that erysipelas is an acutely inflammatory complaint, and must accordingly be treated by extensive depletion; whilst another steps forward and claims the title of this complaint to be classed with morbid poisons, and therefore that precisely the opposite treatment should be adopted; that to bleed is to destroy your patient. We say to both these classes of practitioners alike: attend to symptoms; attend to the predisposing and exciting causes of the disease; attend to the powers and general diathesis of your patient, to the locality and other circumstances of the attack, and then decide on your plan of treatment; instead of coming to the bedside of your patient, disclaiming all the while the influence of preconceived hypothesis, with the determination of treating him on the antiphlogistic or stimulant plan; then, and not till then, may you expect that success which the careful and patient observation and treatment of symptoms alone can justify you in anticipating. Amongst the strenuous advocates of the antiphlogistic treatment we may class Dupuytren and Lawrence, their practice being founded on the opinion that erysipelas is a simple inflammatory affection, and is therefore to be handled like other inflammations, the symptoms, causes, and effects of which are analogous or identical; thus, venesection, local bloodletting, purging, and low diet are the first measures, to which saline and diaphoretic medicines may be afterwards added. "The earlier," says Mr. Lawrence, "these measures are used the better; vigorous treatment in the beginning seems most calculated to shorten the attack, and prevent the disease from spreading beyond its original seat." We now turn for a moment to the supporters of the tonic and stimulant plan of treatment, before we pass on to an analysis of the opinions contained in the works which form the more immediate subject of this review; Dr. Williams, of St. Thomas' Hospital, will afford us the means of exemplifying the theory (not quite peculiar to him) on which this practice is founded. The fairest way of proceeding will be to allow the author to speak for himself:*

"Inflammation may be divided into two great classes, or into those which depend on mechanical or chemical causes, and into those which depend on the agency of morbid poisons. Now a long experience, as well as many experiments, has shown that these different classes of inflammation require the most opposite modes of treatment. The first class which embraces the phlegmasiæ, very constantly requires that the powers of the constitution be brought down to an equilibrium with the state of the part; so that large bleedings are sometimes necessary to induce the inflammation to terminate and resolve, or the wound to heal. The second class embraces a great variety of diseases, as paludal, typhus, and exanthematous fevers, &c., and also erysipelas. In these cases the constitution has very generally received a considerable shock or depression before the tissue, the immediate seat of inflammation, is affected; and consequently the state of the constitution is often at once reduced either to an equality of power with the state of the part, or else below it. And a long experience has shown, that bleeding in this latter class of disease aggravates, as a general principle, all the symptoms, increases the inflammation, and lays the system only the more absolutely under the influence of the poison."

Dr. Williams then proceeds to show that erysipelas depends on the agency of a morbid poison, and is a contagious and infectious disease; and, before developing his own plan of treatment, he criticises rather severely the practice of the two eminent surgeons already mentioned; proving by their own cases, as related by themselves, that their success was anything but uniform, and supporting his objection to bleeding by the well-known experiment of Magendie.* Dr. Williams, likewise, advances in favor of the treatment he adopts, the names of Fordyce, Wells, Heberden, Willan, and even John Hunter; but we are disposed to question whether any of these eminent practitioners (certainly not the last) would have advocated the indiscriminate employment of bark or wine in all cases of true erysipelas. The simple practice of Dr. Williams we again quote from the same source as before:

"The patient is put on a milk diet, the bowels gently opened, and from four to six ounces of port wine, together with sago, allowed daily. This mode of treatment it is seldom necessary to vary throughout the whole course of the dis-

---

*The succeeding extract is taken from the St. Thomas' Hospital Report, vol. i. p. 323. Clinical Observations on Idiopathic Erysipelas, by Dr. Williams.

*Of two rabbits; one was bled largely and both were poisoned by strychnia: the unbled animal survived the other twenty-five minutes.

ease, and the delirium, if present, is generally tranquillised, and, if absent, prevented. The tongue rarely becomes brown, or only continues so for a few hours, and the local disease seldom passes into suppuration or gangrene. In a word, all the symptoms are mitigated, and the course of the disease shortened. I have pursued this treatment for several years, and I hardly remember a case in which it has not been successful."

We observe that the Doctor's objection to bark is founded on the observation, that the patient did not become convalescent till his tongue had become brown, and, consequently, not until he had sunk into a typhoid state: hence originated the preference given to wine. The cases which precede these clinical observations were all treated in this way, and all terminated favorably.

We will now severally notice the general and local treatment of the severer forms of erysipelas advocated by our authors; one of whom, by the way, we are happy to find neither an exclusive admirer of the stimulant or antiphlogistic systems. Mr. Liston remarks, "In severe cases, more especially of phlegmonous erysipelas,* in which there is acceleration of the pulse, and a degree of febrile excitement, general bleeding may be had recourse to; but it must be employed with caution, for the symptoms of increased vascular action may arise from constitutional irritation, and not be meliorated by the depletion." Again, "The exhibition of the extract of aconite in this and other inflammatory affections is often followed by great abatement of vascular excitement, so that the necessity for the abstraction of blood is done away with. It may be given in doses of half a grain in substance, or dissolved in pure water, repeated every third or fourth hour." M. Vidal places the greatest reliance on ipecacuanha, by which, he says, he has arrested the disease where even the cerebral symptoms had become very decided, and that, too, without having recourse to the lancet; but he admits that he has seen the most marked success follow the abstraction of blood frequently repeated, as practised by M. Bouillard, and advocated also by M. Rayer. This latter physician takes the buffy state of the blood as a guide for repetition of the bleeding, and remarks, that when "erysipelas is complicated with phlebitis, the practice ought to be even more energetic." M. Rayer has also found that bathing the parts with cold mucilaginous lotions has been grateful and beneficial, and that "local bleeding, practised at a certain distance from the limits of the inflammation, secures the good effects of the general depletion." (p. 131.) On the other hand Mr. Liston remarks, "In very many cases, the strength is from the first to be supported by all possible means, by nourishing diet, by the exhibition of wine, quinine, and other tonics; more particularly in old people, in constitutions debilitated by disease, in unhealthy situations, and when the fever is of the typhoid kind." The practice of Chelius is likewise founded on the just principles of treating symptoms; freely employing leeches, and even general bloodletting, when the case is urgent: as a local application in his "true" erysipelas, he prefers, in opposition to Rayer, dry warmth. Bleeding by leeches Mr. Liston objects to, on account of the leech bites proving a source of irritation, and being liable to suppurate—a difficulty in a great measure obviated by M. Rayer's plan of placing them beyond the limit of the inflamed surface. In quitting the subject of the general treatment, we must notice a remark of an experienced and practical surgeon (Mr. Pearson,) who considers that cases very rarely occur in large towns in which the practice of bleeding is admissible, and that the repetition of the abstraction of blood should never be had recourse to except under urgent circumstances.* We perceive that Mr. Cooper questions the propriety of making this distinction; it is true that we ought sooner to be guided by "the violence and extent of the inflammation, the state of the pulse and other symptoms, never forgetting the patient's age, strength, and other important considerations," yet we are much disposed to agree with Mr. Pearson, ascribing the different susceptibility and powers of the poor in town and country rather to the influence of their habits and relative use (or rather abuse) of intoxicating liquors, than to locality. We still believe, nay, we are satisfied, that very active depletion has alone sufficed to combat the destructive tendency of this disease, even in the most crowded parts of our metropolis.

We have already noticed the employment of warm and cold applications to a part affected with erysipelas, and we cannot but coincide with Mr. Liston in expressing our opinion that

* As an accurate and precise understanding of what is or ought to be meant by the word "phlegmon" is desirable, we will give in Mr. Lawrence's words his definition of the distinction between it and simple erysipelas; his description differs in no essential from that of Mr. Hunter: "The most striking and important distinctiou between the two affections is, that inflammation is confined to one spot in phlegmon, and is distinctly circumscribed in its seat, while it is diffused in erysipelas, and spreads without limit, This influence seems to depend on the adhesive character of the inflammatory process in the formor; the substance called coagulating, coagulable, or organizable lymph, effused around the inflamed part, forms a boundary between it and the sound portion, which is altogether wanting in erysipelas. In the latter, the effusion is serous; hence, when matter is formed, it is not confined to one spot, but becomes extensively diffused in the cellular tissue."—Med. Chir. Trans. vol. xiv. p. 17.

* Principles of Surgery, p. 211.

the use of spirituous and evaporating lotions, though they may, in many cases, afford temporary relief, "is fraught with the utmost danger; for their direct tendency is to produce metastasis, and if that be to an internal organ of importance, the result is too generally fatal. In case," he adds, "of the translation of erysipelas to any important part, blisters may be applied to the surface which it has left, or to any other in the neighborhood, with the view of recalling the disease to its original and less dangerous situation." (p. 65.) The topical employment of nitrate of silver is a remedy which has been strongly recommended by many surgeons, especially by Mr. Higginbottom: by some the application is confined to the healthy skin immediately around the affected part, by others the whole surface is pencilled over; by the former plan the spread of the inflammation is sometimes arrested, and in the latter, as we have already remarked, it is admissible so long as the inflammation is simply erythematous; but Mr. Liston again warns us against the use of this topical application for the same reason that he eschews cold lotions: namely, the risk of metastasis; and, moreover, he justly remarks, that the inflammation may be extending deeply without our possessing the same facility of detecting its progress when the surface is altered by the action of the caustic. M. Vidal seems adverse to the employment of these local remedies; for he says he has employed, or seen employed, actual cautery, caustic, and pure creosote, without witnessing the beneficial effects described by others. M. Velpeau has proposed to treat erysipelas by compression with bandages, but it does not appear to have been attended with much success in the hands of other practitioners; for we find Chelius, Vidal, Cooper, Lawrence, and others, speaking doubtfully or disparagingly of its effects. The last topical form of treatment we shall notice is that of relief with the lancet or knife; the former consisting in simply puncturing the affected part in numerous places with a fine lancet, the latter having for its object the liberation of distended tissues in the more advanced and acuter forms of the disease: these plans were severally originated and recommended by Sir R. Dobson and Mr. Copland Hutchinson. So much has been said upon the relative merits of the simple puncture and free incision, that we are loath to enter the lists again in deprecating either system exclusively, and in pointing out the propriety of attending to all the symptoms which may be present before any determination is arrived at, regarding the proper course to be pursued. So long as the object is solely to relieve the tension of parts from the abundant serous effusion, or to abstract blood locally, puncturing will be found not only sufficient, but quite as efficacious as larger incised wounds; for the free communication between the cells of the cellular membrane allows of the ready escape of fluid from punctured apertures: but where suppuration and sloughing of the cellular tissue have ensued, as is not unfrequent in the severer forms of phlegmonous inflammation, no doubt can exist regarding the propriety of freely incising the part, although not quite to the extent of sending the patient, as Mr. Cooper remarks, "out of the world in a very sudden manner, sometimes from the shock of an enormous wound on the constitution in its disturbed state, sometimes from profuse hæmorrhage." We say, then, that the actual condition of the affected parts, as ascertained from a history of the progress of the disease and the existing symptoms, must be the chief guide in determining the proper local treatment; and here again we find Mr. Liston's remarks so very practical and apposite that we cannot forbear quoting them:

"It should be borne in mind that the surface of the skin only may be affected—that and the subjacent tissue may be involved, gorged with serous, lymphatic, or purulent infiltration—there may exist great tension of the parts, with a sloughy state of the cellular tissue, established in addition to suppuration; and again, there may be infiltration of the subfascial and intermuscular tissues, leading ultimately to exposure and exfoliation of bones or disease of articulations. From inattention to these circumstances, the treatment being often directed to the name of the disease, great discrepancy of opinion, as to the most proper local management, has arisen."

Mr. Liston adopts free incisions where the tension is great, and there is "a degree of bogginess" marking the disorganized state of the tissues; the relief he ascribes to "abatement of the tension, the unloading of the over-distended blood-vessels of the part, and the acceleration of the suppurative process, which is often critical." The experience of both Chelius and Vidal leads them to confirm this practice, and the latter does not hesitate to recommend the further division of fasciæ when necessary; a step which is sometimes necessary, as we have ourselves proved, when the suppuration is subfascial. Rayer approves of the practice of making incisions, deeming it advisable, in some instances, for the relief of tension, even where there is no suspicion of suppuration having taken place.

We now conclude for the present, designing to avail ourselves of an early opportunity of again meeting our authors and confronting them in the discussion of other divisions of their works, such as the "diseases and injuries of blood-vessels, bones, and joints," which will afford us ample material for a future article.

Of the relative merits of the books themselves we need add but little; for the passing opinion we have at different times expressed, together with the quotations selected, may suffice to put our readers in possession of a fair idea of their comparative value. The work of

M. Vidal certainly introduces us to an author of no ordinary merit; as a surgical treatise it is perhaps to be regarded as the most complete of those before us, but for practical value to the student we cannot but award the preference to the German and English Manuals. On Mr. Liston's work we cannot well bestow more commendation than it deserves; it bears the impress of a practical mind which has long been habituated to accurate observation, and the formation of just and independent conclusions, unbiassed by prejudice or favorite theory, and unencumbered by hypothetical reasoning. The soundness of practice inculcated, and the simple and terse style in which it is set forth, are such as must win the attention and instruct the mind, whilst they tend to infuse self-confidence into the learner.—*Brit. & For. Med. Rev.*

---

*Nerves of the Uterus.*—Dr. Lee has just published some interesting dissections of the nerves of the gravid uterus. After recapitulating the state of our knowledge upon this point at the time he undertook his researches, the British and Foreign Medical Review gives the following abstract.

Each of the hypogastric plexuses, after giving off several branches to the ureter, rectum, and uterus, descends to the side of the neck of the uterus and terminates in a large oblong ganglion. This ganglion receives communicating branches from the sacral nerves, chiefly the third. From the inner surface of the ganglion, numerous small white nerves are given off to the neck of the uterus, some of which ramify under the peritoneum, and others pass deep into the muscular coat. From the anterior and inferior borders of the ganglion, many large nerves are given off to the bladder and vagina, and from its posterior margin to the rectum.—The nerves which come off from the hypogastric plexus before the termination of the latter in the ganglion at the cervix uteri, form a plexus around the ureter and uterine vessels, and prolongations of this plexus pass both to the anterior and posterior surface of the body of the uterus, and are then continued into peculiar membrani form plexuses, with which numerous branches from the ganglia at the neck of the uterus, and from the spermatic plexuses, may be distinctly observed to be continuous. In regard to these plexuses, Dr. Lee remarks:

"From the form, colour, and general appearance of these plexuses on the body of the uterus, and the resemblance they bear to ganglionic plexuses of nerves, and from their branches actually anastomosing and coalescing with the hypogastric and spermatic nerves, I was induced to conclude, on first discovering them, that they were nervous plexuses and constituted the special nervous system of the uterus." (p. 6.)

The following is Dr. Lee's account of the nerves of the gravid uterus in the fourth month' as recorded in his fifth dissection.

"In October, 1840, I finished the dissection of a gravid uterus of four months, all the arteries and veins on the right side of which are completely filled with red and blue injection; and the whole nervous system of the uterus more perfectly displayed than in any of the preparations already described. The uterus was removed from the body of a woman who died in St. George's Hospital, from an external injury, and the fœtus and its appendages were expelled a few hours before death. The nerves were traced while the uterus was covered with rectified spirit.* An artery of considerable size filled with injection is seen accompanying the hypogastric nerve, and passing along with its branches through the hypogastric plexus to the ganglion at the cervix. In this course, the artery is seen ramifying upon the trunk of the hypogastric nerve, and the most minute branches of the hypogastric plexus. The sacral nerves passing into the ganglion are also accompanied with an artery, which is likewise injected, and which passes through the centre of the ganglion. These nerves are a little smaller than in the uteri of nine months. The ganglion is thick, large, and distinct, of an oblong form, about three quarters of an inch in diameter, and consisting of gray and white matter. From its inferior border, three large bundles or masses of nervous fibres are sent off, which present an appearance resembling the pes anserinus of the portio dura. The posterior of these subdivides into numerous small branches, accompanied with arteries which supply the rectum and back part of the vagina. The middle of these great nerves proceeding from the ganglion, likewise accompanied with arteries, ramifies upon the side of the vagina, and the anterior upon the bladder, around the entrance of the ureter. From the hypogastric plexus, before it enters the ganglion, and from the inner surface of the ganglion, numerous large and small branches of nerves are given off to the neck of the uterus, some of which accompany the blood-vessels towards the fundus, and others spread out under the peritoneum. All these are likewise accompanied by injected arteries.—From the inner border of the ganglion, a broad nervous band is sent off, which passes on the outside of the ureter, and another on the inside, which unite and completely surround the ureter. From these united nervous bands, many large branches are sent to the back part of the bladder, and into the anterior part of the cervix uteri. The course of these branches can be

---

* It is only under spirit that dissections of this kind can be properly made. So long a time is necessarily occupied, that if water were used, the parts would putrefy before the dissection could be completed. To attempt tracing the nerves of such a structure as the uterus except under a clear liquid, would be vain.—Rev.

easily traced by their injected arteries. On the lower and anterior part of the cervix uteri, over the mesial line, there is a thick membranous expansion, into which these nerves enter from both hypogastric plexuses and ganglia. From the sides and upper part of this membrane there are given off innumerable filaments, apparently nervous, which unite on the sides of the uterus, with the nerves accompanying the blood-vessels, and with the spermatic nerves, and some of which pass out with the round ligaments. These are likewise accompanied with minute arteries, as all the nerves are on the right side of the uterus, entering the ganglion and passing out from it. The nerves and ganglion on the left side correspond in appearance with those of the right, and the greater number of the spermatic nerves on both sides accompany the spermatic veins."*

Whether Dr. Lee is correct in considering as nerves all the bands and filaments he has met with in his dissections, and which we believe are faithfully delineated in the plates, it is not competent for us to say, nor is it for any one who has not carefully gone through the investigation of the whole subject himself; a task we are aware of no little pains and labour. In these remarks, we allude particularly to the membraniform plexus under the peritoneum, on the body of the uterus, into which there is an evident continuity of branches from what are indisputably nerves. This structure is evidently the same as that alluded to by Lobstein, in the following quotation which Dr. Lee gives in his monograph on the sympathetic nerve: "Hac occasione," says Lobstein, "monendum esse duco, quod, tunica uteri externa sublata, multæ fibræ occurrunt, quæ vario modo sese decussant, et, ope telæ cellulosæ laxæ, tam inter se, quan substantiæ uterinæ profundiori atque densiori, uniuntur. Hæ fibræ, quorum indolem ignoro, facile pro continuatis nervorum ramulis habentur, a quibus vero, non solum ratione directionis atque crassitiei majoris, sed etiam ratione figuræ suæ magis complatatæ, differunt."

It is evidently also the same structure which is described by Madame Boivin and Professor Duges as "a filamentous tissue between the peritoneum and muscular coat of the uterus, and fibres extending longitudinally 'en avant et en arrière sur la regione médiane du viscère de son corps aux moins,'" (see p. 4,) but which, as Dr. Lee remarks, they have not alluded to as having any connexion with the nerves of the uterus. In farther illustration of the history of this subject, Dr. Lee continues: "Dr. Baly states that he met with the following isolated passage in a paper by Schwann—the flat bands under the peritoneum were similar to those described by Lobstein. 'In the uterus of a woman who had borne a child at the full period of pregnancy, I found, immediately under the peritoneal coat, not muscular fibres with transverse striæ, but long very flat bands of different breadth, which presented very indistinct longitudinal striæ, (Langstreifung.) In some few, I could distinguish a flat, pale nucleus, very much elongated, and containing a smaller corpuscle.'"

We have seen from Dr. Lee's historical review that Dr. William Hunter believed that the nerves of the uterus enlarge during pregnancy, but that Mr. John Hunter distinctly denied this. Dr. Lee's dissections can scarcely leave any doubt that the nerves do enlarge during pregnancy, but as to how this enlargement takes place requires yet to be determined.

That the nerves of the uterus, after having performed their proper functions during gestation and labour, gradually return to the condition in which they are found in the unimpregnated uterus, Dr. Lee thinks is made certain by the following observation:

"On the 27th of June, 1840, I examined the uterus of a woman who died suddenly on the tenth day after delivery. The hypogastric plexus, and those both on the anterior and posterior surfaces of the body of the uterus, were very much reduced in size from what they were observed to be in the uteri of six and nine months." (p. 7.)

Dr. Lee concludes the account of his dissections of the human uterus with the following remarks:

"These dissections prove that the human unimpregnated uterus possesses a great system of nerves, which enlarges with the coats, blood-vessels, and absorbants during pregnancy, and which returns after parturition to its original condition before conception took place. It is chiefly by the influence of these nerves that the uterus performs the varied functions of menstruation, conception, and parturition, and it is solely by their means, that the whole fabric of the nervous system sympathises with the different morbid affections of the uterus. If these nerves of the uterus could not be demonstrated to exist, its physiology and pathology would be completely inexplicable." (p. 8.)

Nine dissections altogether of the nerves of the human uterus, both in the impregnated and the unimpregnated state, are recorded in the work before us; an account of a dissection of the nerves of the uterus in the mare is also given, which will be found to present some interesting analogies.

* "Analogy led me to suspect," says Dr. Lee, (p. 7.) "that many branches of nerves would also be found, on examination, to accompany the veins of the spermatic cord, and to ramify upon their coats. Mr. James Dunn, at St. George's Hospital, undertook, at my request, to ascertain if this were the fact, and in July last he made three preparations, in which the nerves are seen covering the veins as as they pass out of the testicle. It appears from these, that a much greater number of nerves accompany the veins, than the artery in the spermatic cord. The nerves in these preparations, form a great plexus around the veins, and are traced into the testicle."

